# The Q_10_ of in situ microbial soil respiration varies with mean annual temperature, precipitation, pH, and plant cover: a meta-analysis and spatial prediction of Q_10_

**DOI:** 10.1038/s41598-026-45615-w

**Published:** 2026-04-02

**Authors:** Melanie T. Hacopian, Eduardo Misael Choreño-Parra, Lirio Hadomi Aquino De Araujo, Andrea Zhou, Charlotte J. Alster, James T. Randerson, Kathleen K. Treseder

**Affiliations:** 1 Ecology and Evolutionary Biology, University of California, Irvine, Irvine, CA USA; 2 Soil and Physical Sciences, Lincoln University, Lincoln, Canterbury, New Zealand; 3 Earth System Science, University of California, Irvine, Irvine, CA USA

**Keywords:** Q10, Soil respiration, Microbial respiration, Macromolecular rate theory, Temperature sensitivity, Mean annual temperature, Climate sciences, Ecology, Ecology, Environmental sciences, Microbiology

## Abstract

The temperature sensitivity of soil microbial respiration, commonly quantified using the Q_10_ coefficient, is a key parameter in carbon cycle models. Uncovering how environmental factors affect in situ Q_10_ values can therefore provide critical insight into potential shifts in global carbon stocks under climate change. We collected data from previously published field experiments that measured soil microbial respiration across a range of temperatures. We hypothesized that the Q_10_ coefficient of in situ soil microbial respiration would vary based on environmental factors including mean annual temperature (MAT), mean annual precipitation (MAP), plant cover type, pH, soil C:N, and latitude. Linear regression revealed that Q_10_ correlates negatively with MAT and MAP and positively with pH and absolute latitude. Additionally, average Q_10_ varied significantly across different plant cover types; it was highest in mountain grasslands and lowest in tropical moist forests. Variation in microbial Q_10_ across environmental factors may arise from underlying mechanisms such as enzyme kinetics, substrate availability and complexity, and microbial adaptation. To capture patterns in Q_10_ more comprehensively, we developed a multiple linear regression model of Q_10_ based on the most individually significant environmental drivers and applied it to public datasets to generate a global map of predicted Q_10_. Q_10_ was higher in high-latitude and high-altitude regions, where large permafrost carbon stores are vulnerable to thawing and decomposition. We also compared fits between the Q_10_ equation and a model produced from macromolecular rate theory (MMRT). We found that the MMRT model had the superior fit and may be better suited to model temperature sensitivity of complex biological reactions. Overall, our results emphasize that relationships between microbial Q_10_ and environmental variables should be accounted for in climate models. Incorporating these variations in the Q_10_ parameter, rather than using a fixed value, will help predict whether CO_2_ emissions will be buffered or exacerbated by soil microbial respiration under climate change.

**Supplementary Information:**

The online version contains supplementary material available at 10.1038/s41598-026-45615-w.

## Introduction

Soil microbial respiration – the release of CO_2_ gas from soil as free-living microorganisms decompose organic matter – is a major component of the global carbon cycle and may contribute to positive feedback to climate warming^1–5^. The temperature sensitivity of this process is often quantified using the Q_10_ coefficient, a metric describing the change in respiration rate given a 10 °C increase in temperature. Understanding relationships between the Q_10_ of soil microbial respiration and environmental factors such as vegetation, temperature, and precipitation is critical to refine climate models, since soil respiration releases about 60 peta-grams (Pg) of carbon into the atmosphere annually^6–8^. Yet, meta-analysis and large-scale soil surveys that examine soil microbial respiration and Q_10_ are rare and often focus on one or two variables of interest^9,10^. To address this gap, we synthesized data from published field studies and analyzed the variability of microbial Q_10_ across several environmental factors. We also generated a geospatial prediction of Q_10_ based on statistically significant environmental drivers.

Soil microbial respiration is temperature sensitive due to the influence of temperature on factors including enzyme kinetics, substrate availability and composition, and microbial community composition^3,11–15^. The temperature sensitivity of microbial decomposition, which underlies rates of soil microbial respiration, is partly intrinsic, determined by the ambient temperature and the complexity of the substrate being decomposed^3,16^. The apparent temperature sensitivity, or the temperature sensitivity that is observed in nature, is the product of the intrinsic temperature sensitivity and any further constraint caused by environmental factors^3^. Understanding how environmental variables affect the temperature sensitivity of soil microbial respiration, and incorporating these relationships into climate models, is critical to accurately predict soil carbon feedbacks to climate change^17^. A key implication of intrinsic temperature sensitivity is that warming in generally colder regions, where decomposition rates are strongly limited by temperature, could lead to disproportionally large increases in CO_2_ emissions^3,10,18^. This concern is particularly acute in Arctic and sub-Arctic regions, where permafrost soils store ~ 1000 Pg of carbon^19–21^. Moreover, these regions are warming two to four times faster than the global average owing to Arctic amplification^22–25^. Thawing permafrost exposes stored carbon to microbial decomposers, but the rate and timescale at which the newly available carbon will be decomposed remains uncertain^26–29^. On the other hand, microbes in warmer regions may already be decomposing near their thermal optimum so any further increase in temperature may not proportionally increase respiration rate, leading to reduced emissions^30,31^.

The Q_10_ temperature coefficient is the most widely used metric to assess the temperature sensitivity of soil microbial respiration^32–34^. While the Q_10_ can be applied to any process, in the context of soil respiration it quantifies the change in respiration rate in response to a 10 °C change in temperature. Since the process of soil microbial respiration is temperature sensitive, climate modelers often incorporate the Q_10_ coefficient in models used to predict carbon dynamics. However, the Q_10_ parameter in these models is often fixed to approximately two and is not distinguished between autotrophic and heterotrophic (i.e. microbial) respiration^35–37^. Static values do not account for spatial and environmental variability which are known to affect Q_10_^9,38–41^. For example, previous studies demonstrate that mean annual temperature (MAT) and mean annual precipitation (MAP) correlate negatively with Q_10_^10,42^. Additionally, Q_10_ has been shown to vary with plant and soil characteristics including vegetation type and pH^43,44^. In this context, models using a fixed Q_10_ value may oversimplify how respiration rate interacts with climate change, which would lead to inaccurate predictions in soil carbon fluxes^40^. However, even if Q_10_ is allowed to vary, failing to represent how its value changes with temperature can further bias model outcomes with one recent study demonstrating how not accounting for the temperature sensitivity of Q_10_ itself results in an underestimation of soil carbon emissions by ~ 5.5–10.4 Pg by 2100^45^. Notably, the DAYCENT model incorporates a variable Q_10_ for decomposition based on temperature, though temperature is not the only factor known to alter Q_10_^46^. In this context, incorporating dynamic Q_10_ values for microbial and root respiration rates could improve climate projections. However, comprehensive meta-analyses on microbial Q_10_ are rare, as most studies evaluate unpartitioned Q_10_ or focus on one or two specific environmental variables of interest^47–50^.

Some researchers have questioned the Q_10_ equation as the most accurate predictor of temperature sensitivity of soil microbial respiration^51–53^. Researchers argue that the Q_10_ equation’s monotonicity (i.e., predicting temperature sensitivity by a single slope parameter) oversimplifies the complex temperature responses that are observed in biological systems^54^. As a result, researchers have proposed alternative frameworks to improve predictions of temperature sensitivity. One such alternative is the Macromolecular Rate Theory (MMRT) framework, which modifies the Arrhenius equation by parameterizing the change in heat capacity experienced by enzymes, complex macromolecules, during a reaction^55^. Since an enzyme’s heat capacity influences the activation energy of a reaction, the activation energy varies with temperature and should be considered dynamic, rather than constant as assumed by the Arrhenius equation^56^. As such, the MMRT framework — although more complex than most models— may better explain the observed decline in enzyme activity at high temperatures. Researchers have begun using the MMRT equation to model soil processes, with some finding a superior fit when compared to the Arrhenius model^12,53,57–59^. However, robust comparisons between the Q_10_ equation and MMRT model as predictors of soil microbial respiration in situ have yet to be made. As the Q_10_ equation assumes a linear relationship between temperature and respiration, while MMRT predicts a curved response, comparisons between the two could reveal whether the Q_10_ equation is overly simplistic and overestimates respiration under warming scenarios in climate models. Since MMRT accounts for a deceleration in the rate of increase of respiration as temperatures approach the thermal optimum, with respiration rates declining after the optimum is exceeded, there may be a self-regulating mechanism that limits soil carbon loss at warmer temperatures^53,59^. The Q_10_ equation does not account for this mechanism. If the MMRT framework better predicts the temperature sensitivity of soil microbial respiration, adopting it in climate models may be worthwhile.

Here, we synthesized published studies to assess how the Q_10_ of soil microbial respiration is affected by environmental factors including MAT, MAP, latitude, soil pH, and soil C: N. Then, to visualize spatial patterns in temperature sensitivity, we generated a global map of predicted Q_10_ values by applying our model of Q_10_ to publicly available climate and soil data. Lastly, we compared the fit of the Q_10_ model to one produced from MMRT. Our dataset included 104 samples from 77 published studies. We tested two hypotheses. First, we hypothesized that the Q_10_ coefficient of soil microbial respiration is modified by environmental factors, including MAT, MAP and latitude (Hypothesis 1). We predicted Q_10_ decreases as MAT increases since previous studies show temperature sensitivity decreases at higher baseline temperatures^10,53,60^. Consequently, we predicted Q_10_ would increase with increasing absolute latitude since MAT and latitude are inversely correlated^41^. We also expected Q_10_ would decrease with increasing MAP, since heavy precipitation reduces substrate availability and soil oxygen^61^. Second, we hypothesized that the MMRT equation is a better predictor of temperature sensitivity than the Q_10_ equation (Hypothesis 2). Our rationale was based on previous studies showing that the MMRT model provides a superior fit than monotonic models for modeling complex, biologically-driven soil processes^12^.

## Methods

### Selection criteria

**Fig. 1:**
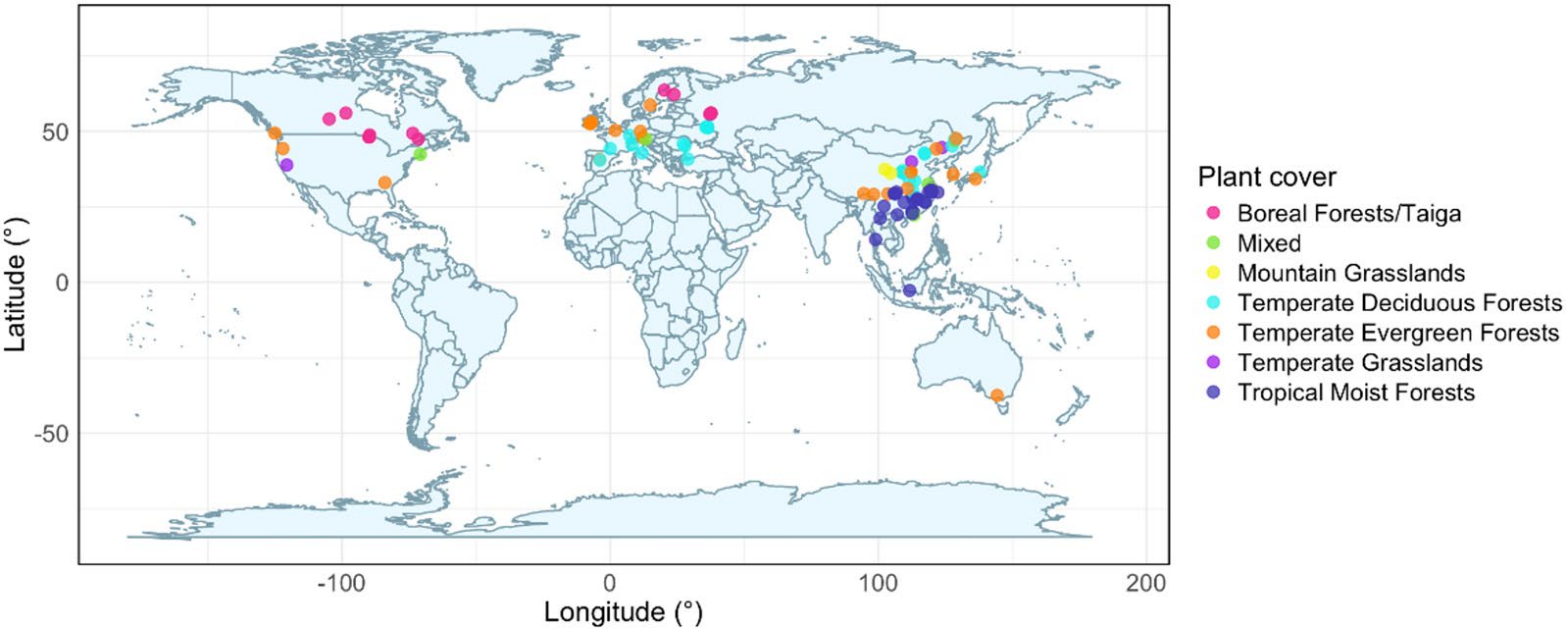
Global distribution of samples used in investigation of the Q_10_ of soil microbial respiration (*n* = 104 samples, from 77 independent studies). Symbol color represents plant cover type. Figure was generated in RStudio (v.4.5.2) using the `ggplot2` and `maps` packages (https://posit.co/download/rstudio-desktop/).

We recorded data from 104 samples in 77 published articles that reported individual rates of soil microbial respiration and corresponding temperatures (Fig. 1). Here, a “sample” refers to a unique site where multiple respiration measurements were made across a range of temperatures. Q_10_ was estimated for each sample from the slope of the log-linear regression between respiration and temperature points. We focused on field experiments but avoided agricultural sites as intensive land management could introduce confounding factors. We conducted a literature search in Google Scholar using as key words “heterotrophic”, “microbial”, “respiration”, “annual”, “partitioning”, and “components”. We also pulled relevant articles from the Soil Respiration Database (SRDB)^62^. The literature search concluded in November 2024 after scanning ~ 660 search results. Respiration rates and temperatures were typically the average of multiple replicates. If studies reported respiration rates and temperatures over multiple years for the same site, we only kept values from the first year. If studies were testing a field manipulation (e.g. drought, warming, fertilizer, etc.) we only kept values from the control or ambient treatment. If publications reported data from multiple sites (i.e. testing the effect of ecosystem type on respiration) we considered each site to be independent. Nevertheless, we accounted for sites stemming from the same publication in our analysis. Studies varied in their approach for partitioning the heterotrophic component of respiration. Most studies employed some method of physical root separation, including trenching, root exclusion collars, and tree girdling. In fewer cases, microbial respiration was determined by equation or substrate induced respiration. Studies also varied in the frequency of measurements and range of temperatures measured (supplemental spreadsheet). We avoided collecting diurnal data, opting for studies that measured respiration on a weekly to monthly basis. The median number of measurements was 12 and the average temperature range was 15.6 °C.

### Data acquisition

In addition to collecting respiration and temperature data for each study, we collected variables that potentially affect the temperature sensitivity of soil microbial respiration. These variables included mean annual temperature (MAT), mean annual precipitation (MAP), absolute latitude, plant cover type, soil pH, soil C: N ratio, as well as minimum and maximum measured soil temperature. In some cases where these values were missing from the original publications, we retrieved them from other studies performed at the same field site.

To extract numerical values from graphs showing respiration or temperature data, we used the digitize package in R (v. 4.4.1)^63,64^. We generated plant cover data using latitude and longitude coordinates and the Global Biomes map layer in ArcGIS (Environmental Systems Research Institute). We crosschecked the ArcGIS output with descriptions and dominant species recorded in the original publication. Any discrepancies were resolved by opting for the biome in ArcGIS that was most closely aligned with the author’s descriptions. After data acquisition, we included 104 samples from 77 published studies (supplemental spreadsheet).

### Q_10_ modeling and analysis

We calculated the Q_10_ coefficient for each sample by fitting the paired, individual observations of temperature and respiration for each study to a log-linear regression model (Eq. 1):

$$\ln \left( {{\boldsymbol{R}}{\boldsymbol{e}}{\boldsymbol{s}}{\boldsymbol{p}}{\boldsymbol{i}}{\boldsymbol{r}}{\boldsymbol{a}}{\boldsymbol{t}}{\boldsymbol{i}}{\boldsymbol{o}}{\boldsymbol{n}}} \right)={\boldsymbol{T}}{\boldsymbol{e}}{\boldsymbol{m}}{\boldsymbol{p}}{\boldsymbol{e}}{\boldsymbol{r}}{\boldsymbol{a}}{\boldsymbol{t}}{\boldsymbol{u}}{\boldsymbol{r}}{\boldsymbol{e}}~ \times ~{\boldsymbol{\beta}_1}+{\boldsymbol{\beta}_0}~$$

β_1_ represents the slope of the regression, β_0_ represents the intercept, and ln is the natural logarithm. We then calculated Q_10_ using the slope value in the following equation (Eq. 2):

$${{\boldsymbol{Q}}_{10}}={{\boldsymbol{e}}^{10 \times {\boldsymbol{\beta}_1}}}$$

We performed mixed, weighted hierarchical regressions to evaluate the effect of climate and soil variables on Q_10_ using the lme4 (v.1.1–35.5) and lmerTest (v.3.1–3) packages in R^65,66^. Since the Q_10_ is fundamentally dependent on the temperature range over which it is measured, we also examined the effect of the minimum, maximum, and range of measurement temperatures collected from each study. Our model followed the formula: Q_10_ ~ variable + (1|Study), weights = *R*^*2*^. Q_10_ was the dependent variable and “Study” is a random factor to account for samples stemming from the same publication. Each continuous variable was a fixed effect. To increase the contribution of better fitted relationships in subsequent analysis, we weighted Q_10_s based on *R*^*2*^ values derived from Eq. 1. The *R*^*2*^ values were not correlated with the number of measurements included in each Q_10_ calculation (Pearson’s *P* = 0.883). We reported both marginal (*r*_*m*_) and conditional (*r*_*c*_) R values, where *r*_*m*_ reflects the variance explained by only the fixed effect and *r*_*c*_ reflects the variance explained by both the fixed and random effects. For plant cover, we performed a linear mixed effects model following the same formula. We then performed a type III Analysis of Variance (ANOVA) on our model following Satterwaithe’s method using the `anova` function from the `stats` package in R (v.3.6.2). We followed up with pairwise t-tests for each pair of plant cover types and used Bonferroni’s method to adjust *P* values. We also analyzed interactions between plant cover and MAT, MAP, and pH by performing separate linear mixed effects models following the same formula but including the plant cover * variable interaction. We then performed a type III ANOVA and conducted post-hoc estimated marginal trend analyses to examine the effect of environmental variables on the Q_10_ within each plant cover type^67^.

### Multiple linear regression and spatial prediction of Q_10_ from environmental drivers

To predict spatial variation of Q_10_, we developed a multiple linear regression model using author-reported Q_10_ values and environmental data derived from public global gridded datasets at the coordinates reported in the author studies. We focused on MAT, MAP, and pH since these factors individually influenced Q_10_ in our meta-analysis. Latitude was not included since its effects are largely covariate with climate and soil properties. Importantly, MAT, MAP, and pH correlated significantly with one another which can potentially lead to inflated standard error and inaccurate model parameterization^68,69^ (Table S1). Additionally, including interaction terms did not improve the variance explained by the model. Accordingly, we chose not to include interactions in our model. We retrieved 30 years (1995–2024) of MAT and MAP data from the ERA5-Land monthly averaged reanalysis dataset (Climate Data Store, Fig. S1). Data were imported to R for further analysis. Furthermore, soil pH was obtained from the SoilGrids database via the `geodata` package in R. All rasters were resampled to a resolution of 0.5° x 0.5°. All raster manipulations were performed using the `terra` package. Climate and soil values were extracted at each study coordinate, allowing direct comparisons between author-reported and publicly available estimates. Here, we display Q_10_ predictions based on public datasets, since the public data is consistent with author estimates and is more reproducible (Fig. S2). Predictions of Q_10_ based on author reported climate and soil values are provided in the supplement.

We fit a linear model following the formula (Eq. 3):

$${{\boldsymbol{Q}}_{10}}=~{\beta _0}+~({\beta _{MAT}}~ \times ~{\boldsymbol{M}}{\boldsymbol{A}}{\boldsymbol{T}})+({\beta _{MAP}}~ \times ~{\boldsymbol{M}}{\boldsymbol{A}}{\boldsymbol{P}})+~({\beta _{pH}}~ \times ~{\boldsymbol{p}}{\boldsymbol{H}})~$$

Here, β_0_ represents the combined intercept and the other β terms represent regression coefficients estimated from each factors data. Coefficients from this model were applied to a global raster via raster algebra (i.e., application of the regression equation to each grid-cell) to generate spatial predictions of Q_10_. Predicted Q_10_ maps were visualized using ggplot2 with observed Q_10_ values overlaid as circle symbols.

### Macromolecular rate theory and Q_10_ comparison

We also modeled microbial respiration using macromolecular rate theory (MMRT). MMRT is a modified version of the Arrhenius equation that accounts for the dynamic heat capacities of enzymes. The MMRT equation is given by the following formula:

$$\ln \left( {\boldsymbol{k}} \right)=\ln \left( {\frac{{{{\boldsymbol{k}}_{\boldsymbol{B}}}{\boldsymbol{T}}}}{{\boldsymbol{h}}}} \right) - \frac{{\Delta {{\boldsymbol{H}}_{{\boldsymbol{T}}\boldsymbol{\theta}}}^{\ddag }+\Delta {{\boldsymbol{C}}_{\boldsymbol{P}}}^{\ddag }\left( {{\boldsymbol{T}} - {{\boldsymbol{T}}_{\mathbf{\theta }}}} \right)}}{{{\boldsymbol{R}}{\boldsymbol{T}}}}+\frac{{\Delta {{\boldsymbol{S}}_{{\boldsymbol{T}}\boldsymbol{\theta}}}^{\ddag }+\Delta {{\boldsymbol{C}}_{\boldsymbol{P}}}^{\ddag }\left( {\ln {\boldsymbol{T}} - \ln {{\boldsymbol{T}}_{\mathbf{\theta }}}} \right)}}{{\boldsymbol{R}}}$$

where k represents the rate of the biological process, T is temperature (in Kelvin), T_θ_ is the reference temperature, k_b_ is Boltzmann’s constant (1.3806e-26 kJ/K), h is Planck’s constant (6.6261e-37 J·s), H is enthalpy, R is the universal gas constant (0.008314 kJ/mol K), S is entropy, and ‡ represents the transition state of the reaction. While Q_10_ is calculated from a single parameter, MMRT fits four parameters: T_θ_, ∆C_P_^‡^, ∆H_Tθ_^‡^, and ∆S_Tθ_^‡^. Typically, T_θ_ is fixed to 4–10 °C below the optimal temperature (T_opt_), which is the temperature at which respiration reaches its maximum rate. T_opt_ can be calculated using the following formula:

$${T_{opt}}=\frac{{\Delta {{\boldsymbol{H}}_{{\boldsymbol{T}}\boldsymbol{\theta}}} - \Delta {{\boldsymbol{C}}_{\boldsymbol{P}}}^{\ddag }{{\boldsymbol{T}}_{\mathbf{\theta }}}}}{{ - \Delta {{\boldsymbol{C}}_{\boldsymbol{P}}} - {\boldsymbol{R}}}}~$$

Additionally, ∆H_Tθ_^‡^, and ∆S_Tθ_^‡^ are not fully independent since an increase in enthalpy is typically associated with a decrease in entropy, further simplifying the model^51,70^. In our MMRT model, k was microbial respiration rate. We set T_θ_ to 4 °C below our calculated T_opt_ of 24.81 °C (T_θ_ = 293.8 K). After model fitting, ∆C_P_^‡^ can be used as a response variable to assess temperature sensitivity. ∆C_P_^‡^ represents the difference in heat capacity between the enzyme-substrate complex and enzyme-transition state complex. The magnitude of ∆C_P_^‡^ determines the steepness of the temperature response curve, where more negative values indicate a greater temperature sensitivity.

Since MMRT fits four parameters while Q_10_ fits one, MMRT requires more data to produce accurate estimates. Specifically, respiration should be measured across at least 20 evenly distributed temperatures ideally including T_opt_^58^. Because temperature is uncontrolled in in situ field experiments and the number of measurements varies across studies, many individual samples did not meet the criteria for optimal MMRT fitting. Therefore, to compare model fits between Q_10_ and MMRT, we calculated Akaike Information Criterion (AIC) values for both models using the temperature and respiration values pooled from all samples. AIC evaluates model performance while accounting for the potential of more complex models to overfit data. An AIC value can be calculated from the following equation:

$${\boldsymbol{A}}{\boldsymbol{I}}{\boldsymbol{C}}=~ - 2\log \left( {{\boldsymbol{m}}{\boldsymbol{a}}{\boldsymbol{x}}{\boldsymbol{i}}{\boldsymbol{m}}{\boldsymbol{u}}{\boldsymbol{m}}~{\boldsymbol{l}}{\boldsymbol{i}}{\boldsymbol{k}}{\boldsymbol{e}}{\boldsymbol{l}}{\boldsymbol{i}}{\boldsymbol{h}}{\boldsymbol{o}}{\boldsymbol{o}}{\boldsymbol{d}}} \right)+2\left( {{\boldsymbol{n}}{\boldsymbol{u}}{\boldsymbol{m}}{\boldsymbol{b}}{\boldsymbol{e}}{\boldsymbol{r}}~{\boldsymbol{o}}{\boldsymbol{f}}~{\boldsymbol{p}}{\boldsymbol{a}}{\boldsymbol{r}}{\boldsymbol{a}}{\boldsymbol{m}}{\boldsymbol{e}}{\boldsymbol{t}}{\boldsymbol{e}}{\boldsymbol{r}}{\boldsymbol{s}}} \right)~$$

In the equation, -2log(maximum likelihood) will reward models with a better fit by lowering the AIC value and + 2(number of parameters) adds a penalty based on the number of parameters to increase the AIC value. The model with the lower AIC value has the superior fit.

## Results

### Hypothesis 1: Effect of environmental factors on Q_10_

**Fig. 2:**
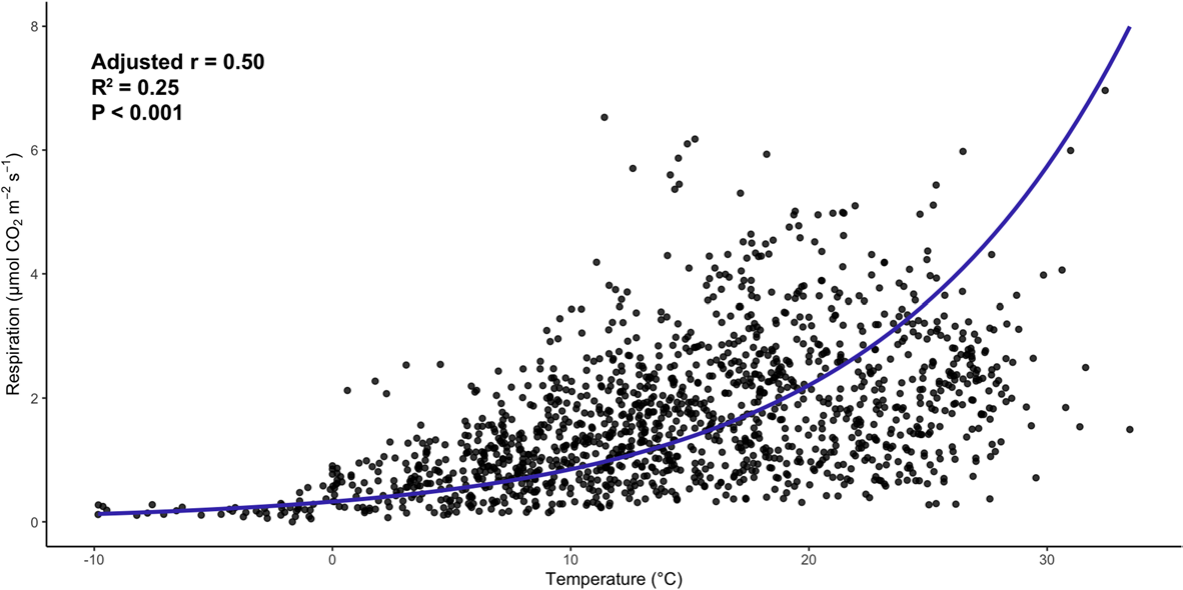
Relationship between soil temperature (°C) and soil microbial respiration (µmol CO_2_ m⁻² s⁻¹) pooled from all samples (*n* = 104). Each symbol represents an observation of average soil microbial respiration at a given average soil temperature (1,434 total observations). The calculated average Q_10_ is 2.60 ± 0.12 SE. Line represents best fit of the regression between temperature and respiration (*r* = 0.50, *P* < 0.001, slope = 0.096).

After compiling 1431 total observations of soil microbial respiration rate from 104 samples, we observed a positive exponential relationship between temperature and respiration (Fig. 2, *r* = 0.50, *P* < 0.001). Accordingly, there was a positive linear relationship between the natural logarithm of respiration and temperature (Fig. S3, *r* = 0.58, *P* < 0.001). When observations were grouped by sample and fitted to the Q_10_ equation, the average Q_10_ was 2.60 ± 0.12 SE.

**Fig. 3:**
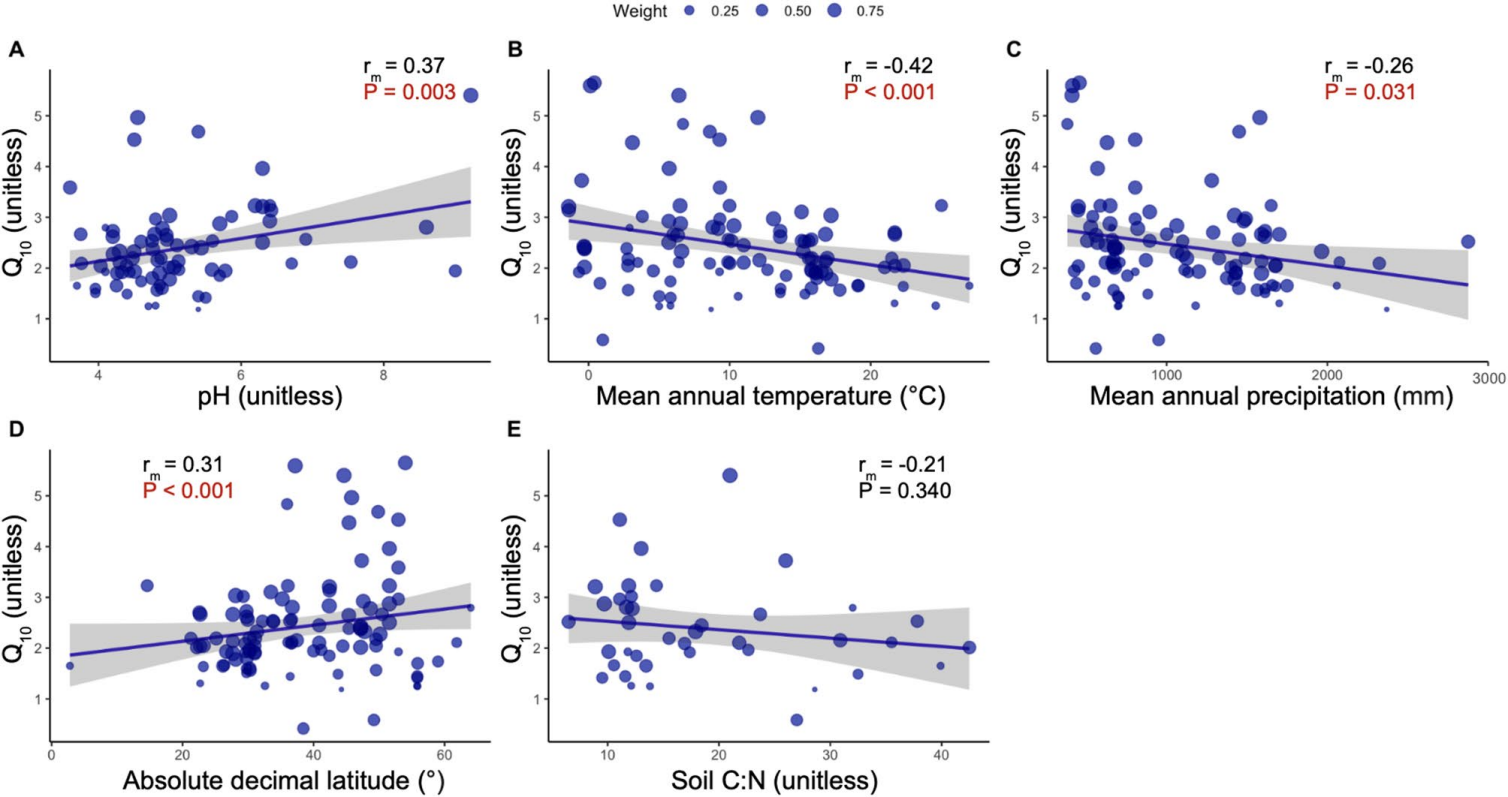
Relationships between the Q_10_ coefficient of soil microbial respiration and pH (A), mean annual temperature (B), mean annual precipitation (C), absolute latitude (D), and soil C: N ratio (E). Each symbol represents a calculated Q_10_ from a sample. Line represents best fit of the linear regression + 95% confidence intervals.

We found that MAT, MAP, pH, and latitude had a significant effect on microbial Q_10_, in support of Hypothesis 1 (Table S2, Fig. 3). Latitude and pH were positively related to Q_10_ (pH *r*_*m*_ = 0.37, Latitude *r*_*m*_ = 0.31). MAT and MAP were negatively correlated with Q_10_ (MAT *r*_*m*_ = 0.42, MAP *r*_*m*_ = 0.26). We did not observe a significant relationship between C: N ratio and Q_10_. Regarding the relationship between Q_10_ and the temperature measurements used in each Q_10_ calculation, we did not observe a significant effect of the minimum measured temperature or temperature range (Table S3). However, Q_10_ was negatively correlated with the mean measured temperature and the maximum measured temperature (Mean temperature *r*_*m*_ = -0.26, maximum temperature *r*_*m*_ = -0.25).

**Fig. 4:**
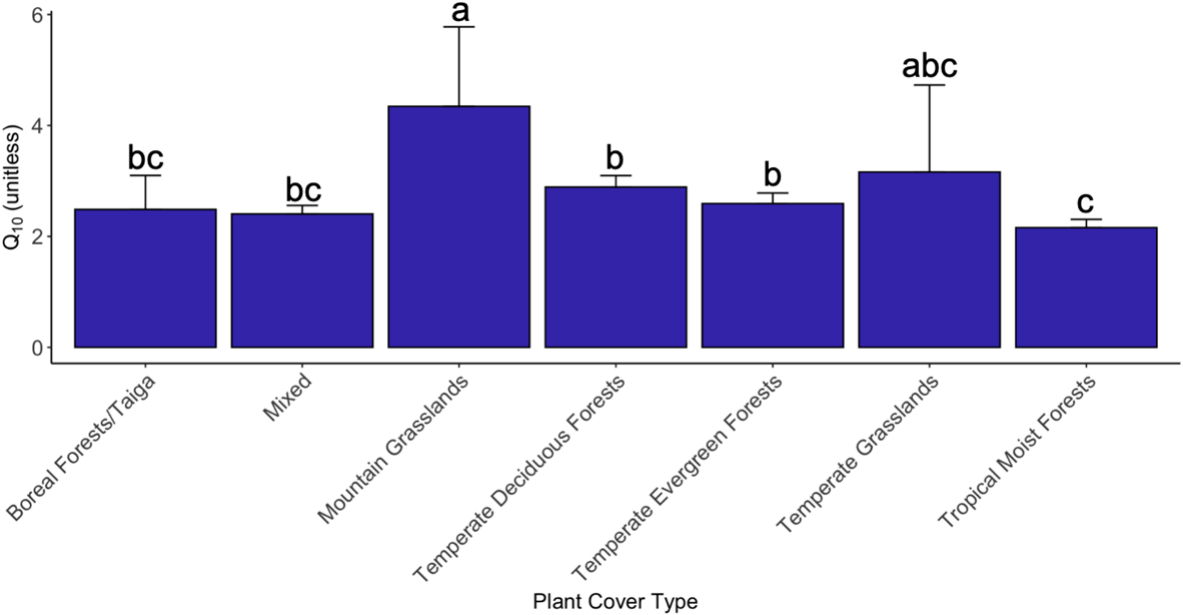
Average Q_10_ of soil microbial respiration across plant cover types (ANOVA *P* = 0.004). Letters indicate statistically significant differences in the Q_10_ value between plant cover types (pairwise *P* < 0.05). Q_10_s were weighted based on *R*^*2*^ values derived from the log-linear regression model between respiration and temperature (Eq. 1). Bars are weighted means ±1SE.

Plant cover also significantly affected Q_10_ (Fig. 4, ANOVA *P* = 0.004). Specifically, Q_10_ was highest in mountain grasslands (Table 1, Q_10_ = 4.35) and lowest in tropical moist forests (Q_10_ = 2.16). Indeed, the Q_10_ in mountain grasslands was significantly higher than all other plant cover types except for temperate grasslands (Table S4, pairwise *P* < 0.05 for all cover types except temperate grasslands). The Q_10_ of tropical moist forests was significantly lower than temperate deciduous forests and temperate evergreen forests (pairwise *P* < 0.001 and *P* = 0.038 respectively). We found significant interactions between plant cover and MAT (*P* = 0.012), MAP (*P* = 0.017), and pH (*P* = 0.021) affecting Q_10_ values. Specifically, MAT had a significantly negative relationship with Q_10_ in boreal forests (Table S5, *P* = 0.001), mountain grasslands (*P* = 0.029), and temperate grasslands (*P* = 0.011). MAP showed the strongest negative relationships with Q_10_ in mountain grasslands (*P* = 0.025) and temperate grasslands (*P* = 0.002). Finally, pH showed a strong positive relationship with Q_10_ in temperate grasslands (*P* = 0.001).

**Table 1:** Characteristics and average Q_10_ values by plant cover type. Q_10_s were weighted based on *R*^*2*^ values derived from the log-linear regression model between respiration and temperature (Equation 1).

Plant cover	*n*	Q_10_	Average mean annual temperature (°C)	Average mean annual precipitation (mm)
Boreal forests / Taiga	13	2.487 ± 0.613*	2.9	715
Mountain grasslands	3	4.345 ± 1.435	5.2	442
Temperate deciduous forests	24	2.891 ± 0.206	7.9	785
Temperate evergreen forests	26	2.593 ± 0.189	9.9	1101
Tropical moist forests	28	2.159 ± 0.149	18.3	1566
Temperate grasslands	3	3.161 ± 1.568	9.1	465
Mixed	7	2.406 ± 0.612	12.3	1279

*Weighted mean ± SE.

### Environmental drivers of Q_10_ and spatial predictions based on public data

Public values of MAT, MAP, and pH were consistent with author reported values (Fig. S2). Accordingly, models of Q_10_ based on public data closely mirrored the author-reported patterns, further supporting Hypothesis 1. Across individual regressions, MAT and MAP were negatively associated with Q_10_ (Table 2, MAT adj. *r* = -0.22, *P* = 0.029, MAP adj. *r* = -0.22, *P* = 0.027). Soil pH showed a significantly positive relationship (Adj. *r* = 0.30, *P* = 0.002). Model performance improved when predictors were combined (Adj. *r* = 0.35, *P* = 0.002).

**Table 2:** Regression statistics for the Q_10_ of in situ microbial soil respiration based on public global-gridded data.

Factor	T	*P*	*r*	*n*
pH	3.18	0.002*	0.30	101
Mean annual temperature (MAT)	-2.22	**0.029**	-0.22	97
Mean annual precipitation (MAP)	-2.24	**0.027**	-0.22	97

Multivariate	*F*	*P*	*r* (multiple)	*r* (adjusted)	*n*
MAT + MAP	3.31	0.051	0.24	0.20	96
MAT + pH	7.79	**< 0.001**	0.39	0.36	93
MAP + pH	6.50	**0.002**	0.35	0.32	93
MAT + MAP + pH	5.35	**0.002**	0.39	0.35	92

*Significant *P*-values in bold.

**Fig. 5:**
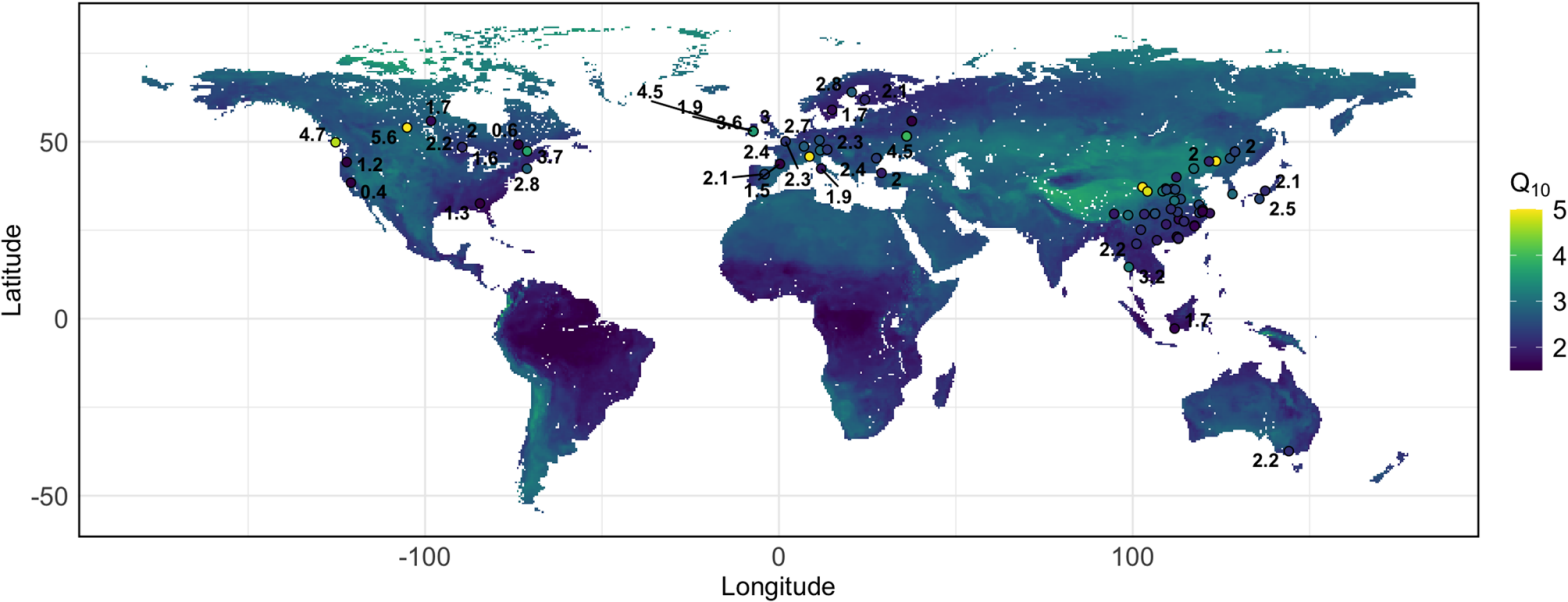
A geospatial prediction of the Q_10_ of soil microbial respiration based on public global gridded datasets. The map shows predicted Q_10_ values generated from a multiple linear regression model incorporating mean annual temperature (MAT), mean annual precipitation (MAP), and soil pH from publicly available datasets (ERA5, SoilGrids). Lighter colors indicate regions with higher predicted temperature sensitivity. White or blank regions indicate large bodies of water. Circle markers represent individual Q_10_ observations from published field studies. Numerical labels for circle markers were only generated if the label would not substantially overlap with other labels or obscure other circle markers. All individual Q_10_ observations with corresponding geographic information are made available in the supplemental spreadsheet.

Applying coefficients from this model to global gridded climate and soil data produced a spatial prediction of Q_10_ that aligned closely with site-level observations (Fig. 5). Broad geographic patters included higher Q_10_s at high latitudes and the Tibetan Plateau. In contrast, Q_10_ was generally lower towards the equator. These geospatial trends were similarly observed in the prediction using author-provided environmental data (Fig. S4). Overall, results indicate that environmental measurements from the public datasets adequately reproduced observed variability in Q_10_.

### Hypothesis 2: MMRT equation and Q_10_ comparison

After fitting pooled global data to the MMRT model, the activation enthalpy (∆H^‡^) was 12.29 kJ/mol. Entropy (∆S^‡^) was − 0.197 J/mol·K and the change in heat capacity (∆C_P_^‡^) was − 3.69 kJ/mol·K. When we compared the fit of the MMRT and Q_10_ models using Akaike’s information criterion (AIC), we calculated an AIC of 3051.47 for the MMRT model and 3139.38 for the Q_10_ model. We predicted that the MMRT model would have a lower AIC value, indicating a superior model fit. Hypothesis 2 was supported, since the MMRT model had a lower AIC value by 89.91 units.

## Discussion

**Fig. 6:**
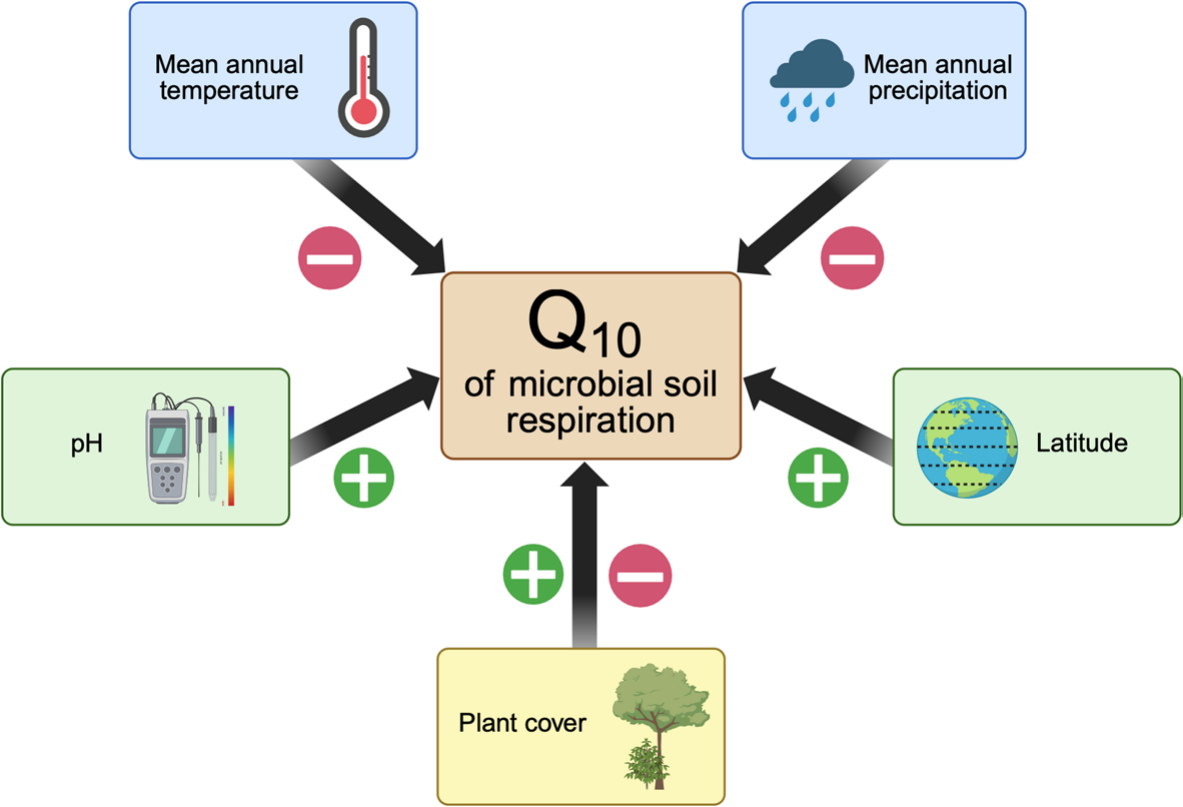
Conceptual figure detailing the effects of environmental variables on Q_10_. We found that the Q_10_ of soil microbial respiration correlates negatively with mean annual temperature and mean annual precipitation and positively with pH and latitude. These relationships could influence soil carbon fluxes under climate change. Created in BioRender. Hacopian, M. (2026) https://BioRender.com/2joh74q.

About 63% of soil respiration stems from microbes, with this number increasing over the years^71^. Here, we focused on how environmental factors modulate the Q_10_ of soil microbial respiration. This information is critical for climate modelers to accurately predict fluxes in soil carbon under future warming^72^. Our study addressed a knowledge gap regarding the relationship between microbial Q_10_ and environmental conditions. We found an average Q_10_ of 2.60 across all samples, greater than the static value of 2 used in many climate models^36,73^. Moreover, microbial Q_10_ correlated negatively with MAT and MAP and positively with latitude and pH (Fig. 6). These trends emerged in geospatial predictions of Q_10,_ regardless of whether author-reported or public measurements of environmental variables were used (Fig. 5, Fig. S4). In addition, Q_10_ varied significantly between plant cover types, and it was the highest in mountain grasslands and lowest in tropical moist forests (Fig. 4). In contrast, Q_10_ did not vary significantly with soil C: N ratio. This finding is consistent with another study that conducted a survey of microbial respiration on 77 unique soils^74^. Additionally, the MMRT framework better predicted temperature sensitivity when we composited data from all samples, supporting Hypothesis 2. Modelers may wish to parameterize microbial Q_10_ to improve the accuracy of soil carbon flux predictions. In addition, the MMRT approach is worth considering.

### Q_10_ is correlated negatively with MAT and MAP, and correlated positively with latitude and pH

The correlations between Q_10_ and MAT, MAP, latitude, and pH provide insight into how soil carbon feedbacks to climate may be influenced by environmental factors. Yet despite the abundance of empirical data, the underlying drivers of temperature sensitivity to decomposition and respiration are highly debated^3,52,75,76^. While it is difficult to tease apart the biological mechanisms behind these correlations, others have suggested that enzyme kinetics, substrate composition and availability, and microbial adaptation and selection play a role^31,49,53,77,78^.

We found that microbial Q_10_ decreased at higher MATs. This finding is consistent with several previous studies that have observed decreases in temperature sensitivity at higher baseline temperatures^9,10,42,49,53,79^. One possible explanation for the decline in temperature sensitivity of soil respiration at higher baseline temperatures is reduced substrate availability^80^. Higher baseline temperatures accelerate microbial decomposition and respiration, which may lead to substrate depletion in warmer soils^31,81–84^. Eventually, this may shift microbial decomposition from being temperature limited to substrate limited^85,86^. If microbial enzymes are already working near their thermal optima at warmer temperatures, but the availability of labile carbon is low due to consistent decomposition, then any further increase in temperature is not likely to drive a strong response. As a result, temperature sensitivity could stagnate at higher temperatures^76,87,88^.

Microbial acclimatization and selection could also contribute to reduced temperature sensitivity at higher temperatures^85,89^. It is well documented that chronic warming alters microbial community composition, biomass, and enzyme production, which in turn affects decomposition, respiration, and temperature sensitivity^31,78,90^. Soil microbes exposed to long term warming have lower temperature sensitivities and respiration rates^31,54,91,92^. Specifically, evidence suggests microbes may acclimatize to warming by downregulating decomposition^93,94^.

We found that microbial Q_10_ of soil respiration decreased as MAP increased, consistent with previous studies evaluating temperature sensitivity and precipitation^10,42,49,95^. One study that collected soil samples across a 2,868 km^2^ area found that increases in soil moisture were associated with decreases in Q_10_, although soil moisture does not always correlate positively with MAP^9^. Heavy precipitation, common in wetlands and peatlands, can affect the apparent temperature sensitivity of soil microbial respiration by lowering oxygen levels in soil^61,96^. When oxygen diffusion declines, aerobic decomposition is inhibited^97^. Subsequently, decomposition by anaerobes is slower owing to less efficient metabolic and respiratory pathways^98–100^. Lack of oxygen can also contribute to the accumulation of decomposition-inhibiting compounds, such as phenols^61^. Furthermore, soil flooding can cause nutrient leaching owing to the downward movement of water. This would lead to substrate limitation in the topsoil where microbial communities are the most active, ultimately causing a decrease in temperature sensitivity^101,102^. In total, our results imply that microbial Q_10_ varies with MAP, which should be accounted for in climate models. If climate models predict reduced precipitation or drought in regions where decomposition was suppressed, there should also be a predicted increase in temperature sensitivity in those regions, translating to greater soil carbon loss^103^.

We also found that microbial Q_10_ correlated positively with absolute latitude. This result is consistent with our findings regarding MAT and MAP. Higher latitudes generally have lower MAT and MAP, which we found leads to increased temperature sensitivity. Other studies have also documented a positive relationship between temperature sensitivity and absolute latitude^10,40,42,49^.

Furthermore, we found a positive correlation between pH and Q_10_ using both author-provided and public values. Specifically, pH showed the strongest positive relationship with Q_10_ in temperate grasslands. Previous research on this relationship is mixed, with evidence of positive, negative, and uncorrelated relationships^43,47,74,95^. Although it has been argued that pH may not have a direct effect on temperature sensitivity, there is evidence for indirect effects, as well as effects arising from pH interacting with other environmental variables^9,47,104^. For example, microbial enzymes involved in decomposition have a pH optimum^105,106^. Evidence shows the optimal pH for microbial activity ranges from five to seven^107,108^. Exceeding the pH optimum may result in slower decomposition and respiration. In other words, any further increase in temperature would not result in a proportional increase in respiration if enzyme ability is hindered. Previous studies report that soil acidification following acid rain led to reduced enzyme activity and lower CO_2_ emissions^47,109^. Additionally, pH can significantly change microbial community composition and reduce biomass, ultimately leading to reduced respiration^106,110,111^. Heavy metals such as iron and aluminum in acidic soils can create a toxic environment and obscure substrates, further limiting respiration^108,111^. Essentially, areas that are predicted to become more acidic may see slower CO_2_ emissions than currently predicted.

### Q_10_ varies across different plant cover types

Plant cover type also significantly affected microbial Q_10_. The highest Q_10_ values were in mountain grasslands (4.36), and the lowest in tropical forests (2.16). Boreal forests, temperate deciduous forests, and temperate evergreen forests shared similar Q_10_ values, which is consistent with other studies^74^. Different biomes typically harbor unique litter and soil characteristics, microbial communities, and climatic conditions, which can all shape how soil microbial respiration responds to temperature^9,41,49,72,95,112^. Additionally, the observed variation in Q_10_ across plant cover types could be a function of soil organic matter quality and availability, which is determined by multiple inputs including plant litter composition^113,114^.

Indeed, researchers have documented that Q_10_ varies considerably across different plant cover and vegetation types^115,116^. For example, Q_10_ in grasslands may be relatively high because grass litter is rich in labile carbon compounds^117^. Previous modeling of Q_10_ across world biomes found that C3 and C4 grasslands had higher Q_10_ values than forests and woodlands^40^. Lower MAT in mountain grasslands may also contribute to higher intrinsic temperature sensitivity, since enzymes must overcome a larger activation energy and work below their thermal optimum. A study surveying Chinese grasslands observed the highest Q_10_ in alpine grasslands, where MAT was lower compared to the other grasslands^118^. Overall, mountain grasslands may exhibit higher Q_10_ values due to colder climate. In contrast, tropical moist forests may display lower Q_10_ values owing to warmer climate and high levels of precipitation^49^. In these regions, increasing temperature may not proportionally accelerate respiration if high baseline decomposition rates deplete substrate availability. Additionally, temperature sensitivity in tropical moist forests could be lowered due to low soil oxygen stemming from high soil moisture.

### Relationships between environmental variables and Q_10_ suggest carbon climate feedback may be underestimated

Overall, our findings have important implications for soil carbon feedbacks to climate warming. In warmer regions, the observed decrease in temperature sensitivity could buffer soil carbon emissions^78^. In colder regions, higher Q_10_s could increase fluxes of CO_2_ emissions as microbial activities increase with higher temperatures and decompose large stores of labile carbon, contributing to positive feedback to warming^10^. This is particularly relevant in Arctic and sub-Arctic permafrost zones, which contain a significant store of soil organic carbon (~ 1000 Pg or one trillion metric tons)^19–21^. Moreover, these regions are particularly prone to warming, with most studies reporting rates of warming that are two to four times faster than the global average^22–29,119^. This phenomenon, termed Arctic amplification, is driven by factors including rising ocean and air temperatures, loss of albedo caused by ice and snow melt, and changes in vegetation^29,120–124^. As permafrost thaws, previously frozen carbon is freed up for decomposition, leading to potentially large releases of greenhouse gases. One study suggests that ~ 119–251 Pg of carbon will become available for decomposition by 2100 owing to permafrost thaw^125^. Our analysis shows that Q_10_ values are higher in high latitude regions, suggesting that the temperature sensitivity of soil microbial respiration is amplified exactly where soil carbon stores are vulnerable (Fig. 5). Consequently, this indicates that current carbon-climate feedback models that do not account for this elevated temperature sensitivity may underestimate future carbon losses under continued warming. However, we suggest that our findings be interpreted with caution. Our dataset was skewed to regions in North America, Europe, and Asia, and lacked studies from the Global South, making extrapolations there potentially less reliable. Moreover, variation in the temperature ranges over which soil microbial respiration was measured across studies likely influenced the Q_10_s we observed and therefore may have introduced some uncertainty. Additionally, caution is warranted in interpreting our predictions as our model explains only a minority of the variance. It is possible that the high complexity and heterogeneity of soil limited predictive performance^126–128^. We acknowledge that other unmeasured variables, such as microbial community composition, may play an important role in shaping soil microbial Q_10_^14,129^. Future studies that integrate microbial, soil, and climatic drivers of soil microbial Q_10_ will be valuable.

### MMRT better predicts temperature sensitivity compared to Q_10_

The MMRT model had the superior fit for our data compared to the Q_10_ model, as evidenced by the lower AIC value. MMRT was first introduced as a model that expands upon the Arrhenius equation by accounting for the temperature sensitivity of enzymes and can explain declines in enzymatic activity before denaturation temperatures^55^. The MMRT equation asserts that changes in the heat capacity of the enzyme-substrate complex during a reaction leads to reduced enzyme efficiency at high temperatures^51^. In contrast, the Arrhenius and Q_10_ models predict that reaction rates will simply increase exponentially. While this pattern may be observed for small inorganic molecules, enzymes are complex macromolecules with large heat capacities^130^. More energy is needed to raise their temperature, and therefore their activation energy is temperature dependent. Our finding is consistent with other studies that have compared the fit of the MMRT model to other linear and log-linear models^12,53^. Our results, in addition to previous comparisons, demonstrate that the temperature sensitivity of soil microbial respiration rates do not follow a linear trend as predicted by the Q_10_ and Arrhenius models. Rather, the slope of the relationship between temperature and soil microbial respiration may depend on the range of temperatures captured. Therefore, the temperature sensitivity of soil microbial respiration may follow a more unimodal curve in which respiration rates peak. Because MMRT accounts for declines in enzyme activity at high temperatures, and the Q_10_ model does not, carbon cycling models that adopt MMRT may predict lower rates of soil carbon loss in warmer regions than would those with the Q_10_ model.

There are challenges in adopting the MMRT framework. The MMRT equation is considerably more complex than Q_10_, requiring four fitted parameters in contrast to only one. For this reason, the MMRT model requires more data to accurately estimate its required parameters. A study by Robinson et al. 2017 found that at least 20 different temperature points should be included in measurements for the optimal MMRT fit^58^. Additionally, T_opt_ should also be included in temperature measurements^70^. Accordingly, we chose not to calculate MMRT parameters for each sample, as many of our samples lacked the number of measurements or broad temperature range that is recommended for reliable results. Instead, we calculated a global MMRT from all measurements combined. Future field experiments that take frequent measurements (e.g. weekly or biweekly) across one or more years would be valuable.

## Conclusion

The fate of global carbon stocks under future warming remains difficult to discern despite an abundance of research. Understanding how the temperature-response of soil microbial respiration—which converts soil carbon to CO_2_—varies with environmental factors is necessary to help answer this question. In summary, we found that the Q_10_ of in situ soil microbial respiration is modulated by MAT, MAP, latitude, and pH using data from 77 studies. Our results demonstrate that soil microbial temperature responses are not spatially uniform; rather, they are modulated by climatic and soil factors in a manner that can influence the storage versus release of carbon from soils. These relationships should be considered to refine climate models predicting future soil carbon fluxes. In already warm regions, further warming may not drive as much of an increase in CO_2_ emissions as currently predicted. In previously cold and wet regions where soil microbial respiration is more temperature sensitive, warming and drying may lead to accelerated decomposition and greater CO_2_ emissions than expected based on a static Q_10_.

## Supplementary Information

Below is the link to the electronic supplementary material.

Supplementary Material 1

Supplementary Material 2

## Data Availability

Code used for this study is deposited at [https://github.com/melaniehacopian/microbial\_Q10\_workflow]. The main dataset including temperature and respiration measurements, author and study descriptions, and study metadata is included in the supplement. Other data will be made available upon request. Please contact the corresponding author (M. T. Hacopian) to request data.
