# Supplementary material for: The Q10 of in situ microbial soil respiration varies with mean annual temperature, precipitation, pH, and plant cover: a meta-analysis and spatial prediction of Q10
Source: Sci Rep. 2026 Apr 2;16:15691. doi: 10.1038/s41598-026-45615-w (PMC13187268; doi:10.1038/s41598-026-45615-w)
Supplement: Supplementary file 2 — Supplementary Material 2 [file 41598_2026_45615_MOESM2_ESM.docx]

**Supplementary Table 1.** Pearson’s correlation table for author provided mean annual temperature (MAT), mean annual precipitation (MAP), and pH.

| Factor(s) | pH  *(r, P)* | Mean annual temperature  *(r, P)* | Mean annual precipitation  *(r, P)* |
| --- | --- | --- | --- |
| pH | 1.0, NA | **-0.30, 0.010** | **-0.46, <0.001** |
| Mean annual temperature (MAT) | **-0.30, 0.010** | 1.0, NA | **0.61, <0.001** |
| Mean annual precipitation (MAP) | **-0.46, <0.001** | **0.61, <0.001** | 1.0, NA |

*Significant *P*-values in bold.

**Supplementary Table 2.** Regression statistics for the Q_10_ of *in situ* microbial soil respiration using author-provided environmental variables.

| Factor(s) | *T* | *P* | *r* (marginal) | *r* (conditional) | *n* |
| --- | --- | --- | --- | --- | --- |
| pH | 3.13 | **0.003*** | 0.37 | 0.78 | 75 |
| Mean annual temperature (MAT) | -3.51 | **<0.001** | -0.42 | -0.79 | 102 |
| Mean annual precipitation (MAP) | -2.20 | **0.031** | -0.26 | -0.75 | 101 |
| Absolute latitude | 2.39 | **<0.001** | 0.31 | 0.74 | 104 |
| Soil C:N ratio | -0.97 | 0.340 | 0.21 | 0.21 | 40 |
| **Multivariate** | *F* |  | *r* (multiple) | *r* (adjusted) |  |
| MAT + MAP | 4.51 | **0.013** | 0.29 | 0.26 | 98 |
| MAT + pH | 4.51 | **0.014** | 0.33 | 0.29 | 73 |
| MAP + pH | 4.00 | **0.023** | 0.32 | 0.28 | 71 |
| MAT + MAP + pH | 2.87 | **0.043** | 0.33 | 0.27 | 69 |

*Significant *P*-values in bold.

**Supplementary Table 3.** Regression statistics for the Q_10_ of *in situ* microbial soil respiration and the minimum, maximum, mean, and range of measurement temperatures reported by each study.

| Factor(s) | *T* | *P* | *r* (marginal) | *r* (conditional) |
| --- | --- | --- | --- | --- |
| Minimum temperature | -1.31 | 0.192 | -0.16 | -0.76 |
| Maximum temperature | -2.07 | **0.042** | -0.25 | -0.72 |
| Mean temperature | -2.02 | **0.031** | -0.26 | -0.74 |
| Temperature range | -0.37 | 0.712 | -0.04 | -0.76 |

*Significant *P*-values in bold.

**Supplementary Table 4.** Pairwise t-test comparisons on Q_10_ of microbial soil respiration across different plant cover types. *P* values were adjusted using the Bonferroni method.

| Comparison | *T* | *P* adjusted |
| --- | --- | --- |
| Boreal Forests/Taiga – Mixed | 0.26 | 0.619 |
| Boreal Forests/Taiga – Mountain Grasslands | 7.10 | **0.018*** |
| Boreal Forests/Taiga – Temperate Deciduous Forests | 2.83 | 0.101 |
| Boreal Forests/Taiga – Temperate Evergreen Forests | 0.89 | 0.352 |
| Boreal Forests/Taiga – Temperate Grasslands | 0.21 | 0.654 |
| Boreal Forests/Taiga – Tropical Moist Forests | 0.06 | 0.813 |
| Mixed – Mountain Grasslands | 14.18 | **0.006** |
| Mixed – Temperate Deciduous Forests | 1.06 | 0.312 |
| Mixed – Temperate Evergreen Forests | 0.04 | 0.842 |
| Mixed – Temperate Grasslands | 0.05 | 0.833 |
| Mixed – Tropical Moist Forests | 2.73 | 0.108 |
| Mountain Grasslands – Temperate Deciduous Forests | 9.01 | **0.006** |
| Mountain Grasslands – Temperate Evergreen Forests | 13.01 | **0.001** |
| Mountain Grasslands – Temperate Grasslands | 1.16 | 0.342 |
| Mountain Grasslands – Tropical Moist Forests | 40.41 | **<0.001** |
| Temperate Deciduous Forests – Temperate Evergreen Forests | 1.36 | 0.250 |
| Temperate Deciduous Forests – Temperate Grasslands | 0.05 | 0.830 |
| Temperate Deciduous Forests – Tropical Moist Forests | 12.41 | **<0.001** |
| Temperate Evergreen Forests – Temperate Grasslands | 0.04 | 0.840 |
| Temperate Evergreen Forests – Tropical Moist Forests | 4.52 | **0.038** |
| Temperate Grasslands – Tropical Moist Forests | 1.10 | 0.304 |

*Significant *P*-values in bold.

**Supplementary Table 5.** Estimated marginal trend analysis of the effect of MAT, MAP, and pH on the Q_10_ of each plant cover type.

| Plant cover type | Trend (slope) | *t* ratio | *P* |
| --- | --- | --- | --- |
| **Mean annual temperature** |  |  |  |
| Boreal forests / Taiga | -0.52 | -4.01 | **0.001** |
| Mountain grasslands | -0.28 | -2.24 | **0.029** |
| Temperate deciduous forests | -0.02 | -0.73 | 0.466 |
| Temperate evergreen forests | -0.04 | -1.02 | 0.314 |
| Temperate grasslands | -0.27 | -2.68 | **0.011** |
| Tropical moist forests | 0.01 | 0.15 | 0.880 |
| Mixed | -0.03 | -0.67 | 0.503 |
| **Mean annual precipitation** |  |  |  |
| Boreal forests / Taiga | 2.04e-4 | 0.16 | 0.871 |
| Mountain grasslands | -1.81e-2 | -2.31 | **0.025** |
| Temperate deciduous forests | 1.21e-4 | 0.24 | 0.814 |
| Temperate evergreen forests | 5.44e-5 | 0.17 | 0.863 |
| Temperate grasslands | -2.66e-2 | -3.38 | **0.002** |
| Tropical moist forests | 4.10e-5 | 0.03 | 0.973 |
| Mixed | -4.72e-4 | -0.61 | 0.543 |
| **pH** |  |  |  |
| Boreal forests / Taiga | Insufficient pH data |  |  |
| Mountain grasslands | Insufficient pH data |  |  |
| Temperate deciduous forests | 0.22 | 0.91 | 0.367 |
| Temperate evergreen forests | 0.01 | 0.03 | 0.977 |
| Temperate grasslands | 15.72 | 3.55 | **0.001** |
| Tropical moist forests | 0.40 | 1.34 | 0.192 |
| Mixed | 0.43 | 0.86 | 0.394 |

*Significant *P*-values in bold.


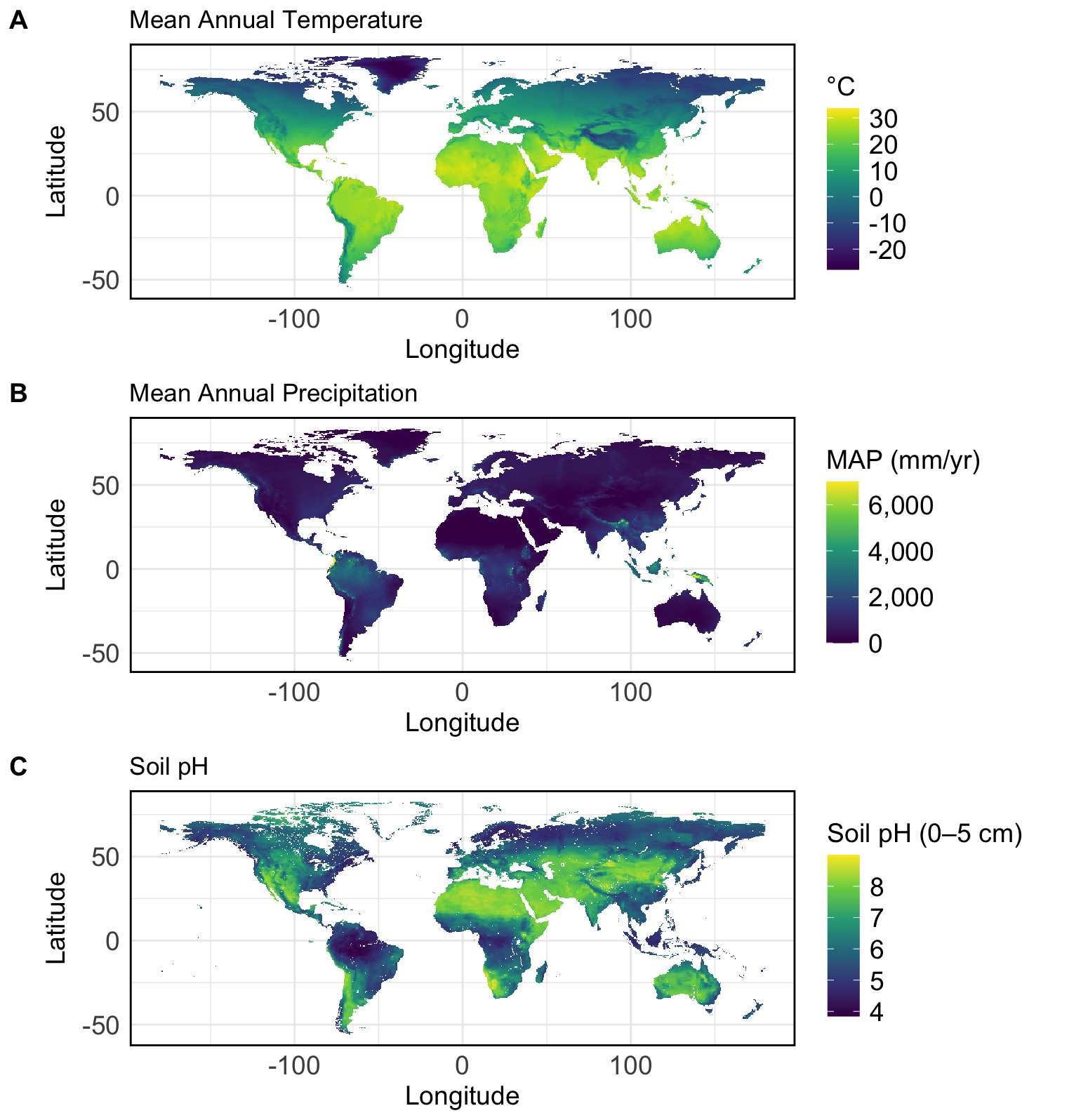


**Supplementary Figure 1.** Global distribution of mean annual temperature, mean annual precipitation, and soil pH retrieved from publicly available databases (ERA5 and SoilGrids).


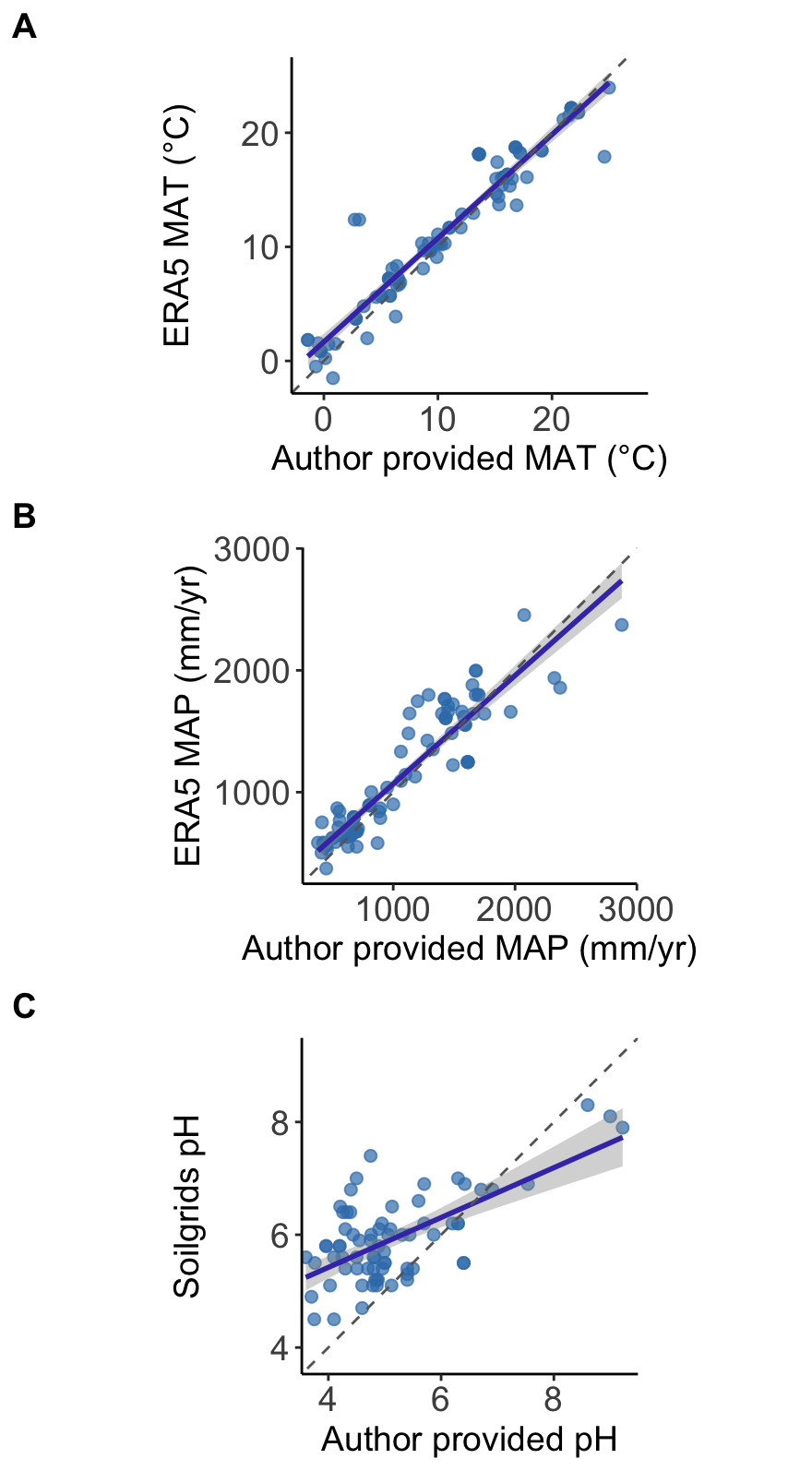


**Supplementary Figure 2.** Line graph comparisons of author-provided and public values. Public values of mean annual temperature (MAT), mean annual precipitation (MAP), and soil pH were crosschecked with author measurements at the same geospatial coordinates. Dashed line represents a slope of 1.

**
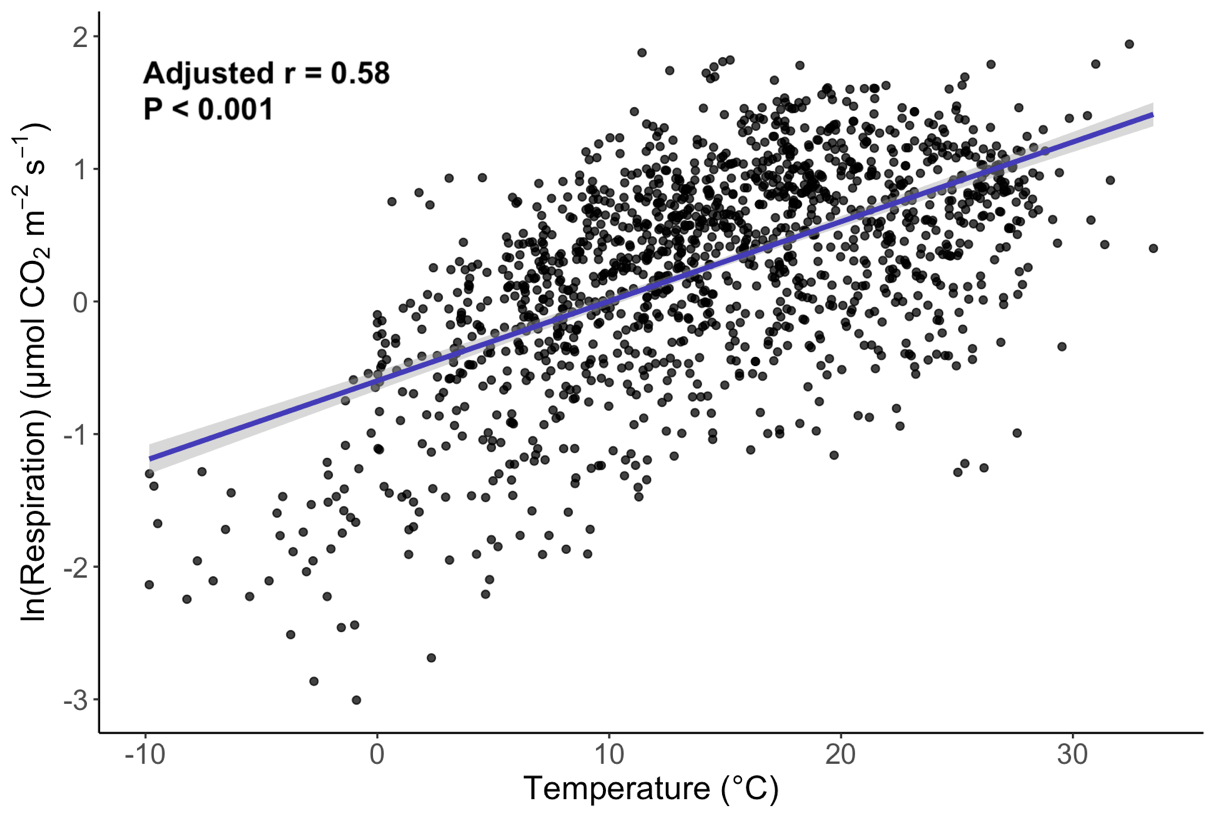
**

**Supplementary Figure 3.** Relationship between the natural logarithm of soil microbial respiration (µmol CO_2_ m⁻² s⁻¹) and temperature (°C) pooled from all samples (*n* = 104). Each symbol represents an observation of soil microbial respiration at a given soil temperature (1,434 total observations).

**
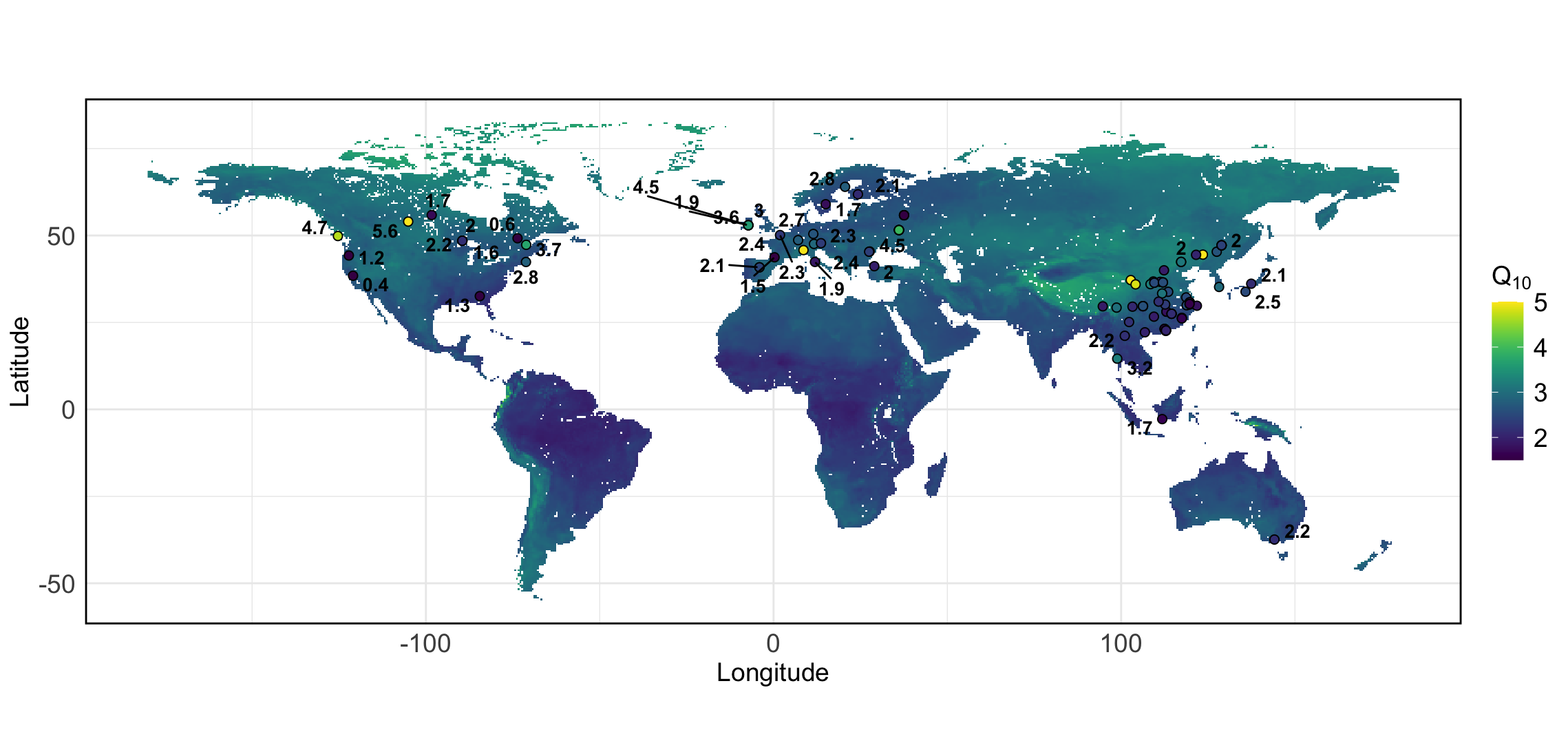
**

**Supplementary Figure 4.** A geospatial prediction of the Q_10_ of microbial soil respiration based on author-reported measurements of environmental variables. The map shows predicted Q_10_ values generated from a multiple linear regression model incorporating mean annual temperature (MAT), mean annual precipitation (MAP), and soil pH reported by the authors. Lighter colors indicate regions with higher predicted temperature sensitivity. Circle markers represent individual Q_10_ observations from published field studies
